# Advanced Dual−Function Hollow Copper−Sulfide−Based Polyimide Composite Window Film Combining Near−Infrared Thermal Shielding and Organic Pollutants’ Photodegradation

**DOI:** 10.3390/polym14163382

**Published:** 2022-08-18

**Authors:** Xiangfu Liu, Jinming Ma, Jiulin Shen, Jianqiao Zhao, Chengxu Lu, Guoli Tu

**Affiliations:** Wuhan National Laboratory for Optoelectronics, Huazhong University of Science and Technology, 1037 Luoyu Road, Wuhan 430074, China

**Keywords:** in situ growth, hollow Cu_2−x_S/PI composite film, dual-function window film, near-infrared thermal shielding, formaldehyde degradation

## Abstract

Window−film−integrated, near−infrared (NIR) absorption−based nanomaterials are of great interest in terms of numerous demands to reduce energy consumption, especially in buildings and vehicles. However, the question of how to effectively manage thermal energy generated from NIR harvesting in light−absorbing materials, rather than being wasted or causing negative effects, remains challenging. Herein, hollow copper sulfide (Cu_2−x_S) on colorless polyimide (PI) films, enabling them to be well−dispersed and robustly adhered, underwent in situ growth fabrication and were utilized as NIR−thermal−shielding and organic−pollutant−removal dual−function window films. Due to strong NIR absorbance, arising from the heavy hole−doping (copper cation deficiency), the Cu_2−x_S/PI composite film exhibited great promise for use in the filtration of the NIR spectrum. By monitoring Cu_2−x_S densities, its NIR−shielding efficiency reached 69.4%, with hundred−percent UV blocking and consistent performance within the reliability (85 °C/85%RH) tests over one week as well as 5000 bending cycles. The integration of the films into model cars and building windows exhibited excellent thermal−shielding performance upon exposure to direct sunlight. Moreover, benefiting from the distinctive distribution of Cu_2−x_S, the additional thermal energy (holes) generated in NIR absorption was successfully utilized. The densely surface−confined hollow structure of Cu_2−x_S on PI significantly endowed good formaldehyde catalytic capacity, with removal efficiency reaching approximately 72% within 60 min and a negligible decline after quartic reuse. These integration methodologies enable the promising fabrication of a high−performance, bifunctional window film combining thermal shielding and indoor organic pollutant removal.

## 1. Introduction

In recent decades, saving energy and purifying the environment are of great significance in buildings and vehicles, where we spend from 80 to 90% of our time. Excessive energy consumption for cooling and heating (air−conditioning) leads to explosive negative effects such as serious environment pollution and global warming [1,2]. In comparison to other components, the thermal capability of glazed components of buildings is far poorer; as much as 50% of the total energy is lost or gained through windows with poor thermal shielding or insulation, which is expected to continue to dramatically increase due to the growing preference for panoramic glass architecture [3,4,5]. To reduce energy consumption, window films integrated with thermal−shielding nanomaterials (including absorption− and reflection−based materials) have garnered intensive attention [6,7,8,9]. Taking into consideration the usage of buildings and vehicles, thermal−shielding nanomaterials require visible transparency and a favorable filter in near−infrared (NIR) radiation because 51% of the solar energy radiation is distributed within the NIR region in the solar spectrum [10]. Numerous materials have been widely investigated for use in blocking NIR radiation, including NIR−reflecting materials such as TiO_2_, BaTiO_3_, Cr−doped BiPO_4_, etc. [11,12,13,14], NIR absorption materials, for instance, metals (Cu and Au), metal oxides (SnO_2_, V_2_O_5_, and WO_3_), as well as the associated material dopants [15,16,17,18,19,20,21,22]. Considering light pollution in reflective materials [23,24], NIR−absorbing materials exhibit more potential in many application scenarios and requirements due to their excellent tunable optical, electrical, and magnetic capacities [25]. However, effectively managing the additional thermal energy generated from NIR harvesting, rather than it being wasted or causing negative effects, remains a challenge in light−absorbing materials.

In addition to thermal shielding, environmental quality management is another important prerequisite to enable individuals to stay indoors for a long time, considering the variety of organic pollutants, especially air pollutants such as second−hand smoke, formaldehyde (HCHO), and harmful substances such as CO, SO_2_, etc., which probably exist in indoor air [26,27,28]. Among these volatile indoor organic pollutants, HCHO has attracted serious attention for its extensive use in decorative materials, wooden buildings and furniture products, etc. The long−term exposure to indoor pollutants containing HCHO is highly deteriorative to human health and potentially carcinogenic [29]. Among various strategies adopted to realize HCHO removal, its photocatalytic oxidation into harmless byproducts is the most attractive method which possesses robust efficiency and low energy consumption. In recent decades, many materials such as MnO_2_, CeO_2_, Co_3_O_4_, and so forth [30,31,32,33,34,35,36,37], have demonstrated potential capacities for use in HCHO catalytic degradation. Thus, the identification of nanomaterials that can be simultaneously used in thermal shielding and photocatalysis to be integrated into window films is important.

Alternatively, the development of nanomaterials that can utilize the energy generated in NIR harvesting for photocatalysis is preferred. Here, the nontoxic material, copper sulfide (Cu_2−x_S), was employed, and it is of interest in numerous fields such as energy and biotechnology [38]. Copper (I) sulfide (Cu_2_S) possesses a tendency to lose cations because of oxidation, and copper has high mobility [39]. The resulting copper deficiency generates free holes in the valence band, rendering it a p−type self−doped material [40,41]. Heavily cation−deficient Cu_2−x_S owns a high hole density (10^21^ cm^−3^) which enables the strong NIR harvesting of localized surface plasmon resonance (LSPR), which gives them great promise in the NIR harvesting of solar radiation for thermal shielding [42,43,44]. Meanwhile, due to its tunable plasmonic absorption, hole−doped Cu_2−x_S is a potential candidate for use in building SPR−enhanced composite photocatalysts, making successful use of the vis−NIR region of the solar spectrum [45,46,47,48,49]. Therefore, integrating Cu_2−x_S into flexible substrates for high−efficiency NIR shielding and utilizing the additional thermal energy from NIR harvesting for indoor HCHO removal for use in dual−function window films is very promising.

To take good advantage of the additional generated heat from NIR absorption for pollutants’ photodegradation, it is necessary to ensure enough nanomaterials are exposed to the surface of a film, so as to offer a sufficient contact area with pollutants for efficient catalytic degradation. Commonly used fabrication procedures for NIR−absorbing materials for window films, which disperse the nanomaterials into a prepared polymeric precursor solution and use surface coating on substrates, or magnetron−sputtered metals and other materials on a flexible substrate with high tension [3], are not feasible at this point, as particles are entirely distributed inside the film in dispersion coating, and magnetron sputtering results in a continuous layer, which cannot offer sufficient reaction sites and contact areas with pollutants. Additionally, surface−exposed nanomaterials cause difficulties in adhesion and dispersion because the brittleness, which detaches and aggregates particles, decreases the LSPR and thus reduces the NIR absorption. Therefore, the question of how to uniformly and firmly integrate Cu_2−x_S into a flexible substrate surface is challenging. Initially, colorless polyimide (PI) film and the in situ growth method were employed in this work. As an ultraviolet (UV) cutoff, PI film is a kind of special material with good thermal stability and flame resistance, which has been extensively employed in many fields [50]; it was previously adopted as a UV−blocking film against the aging of leather furniture and human skin. Additionally, the imide rings in PI chains provide abundant activated sites for the selectivity bonding of external ions after alkali treatment, which enables the possibility of the in situ growth of Cu_2−x_S on a PI surface to form a thermal−shielding film. The direct growth of nanostructures from the molecular chain would increase the adhesion between the two. Moreover, the main benefit of the in situ fabrication technique is the controllable dispersion capability of the surface−confined nanostructures. As a result, hollow Cu_2−x_S was successfully integrated into PI via in situ growth fabrication. These Cu_2−x_S/PI films exhibited high visual transparency and excellent blocking efficiency in both the UV and NIR regions. Benefiting from the distinctive distribution and hollow structure of Cu_2−x_S, the additional thermal energy from their NIR harvesting was successfully utilized for efficient photocatalysis, and a good HCHO catalytic efficiency was achieved by monitoring the Cu_2−x_S densities, indicating that the facile integration method provides a promising fabrication of reliable thermal−shielding and organic−pollutant−removal bifunctional window films.

## 2. Experimental Section

### 2.1. Fabrication of Cu NP Composite PI

The in situ growth of metallic Cu on PI films was achieved as in previous work [51]; typically, to hydrolyze the imide rings in PI chains, the PI was treated with a potassium hydroxide (KOH) aqueous solution (5 M) for 30–300 min. Then, it was moved to a 1 M cupric nitrate (Cu(NO_3_)_2_) aqueous solution to undergo Cu cation exchange for 1 h. Subsequently, the Cu ions were chemically reduced into metallic Cu with a dimethylamine borane (DMAB) aqueous solution (10 mM) for 5 h. After each step, the samples were rinsed with abundant DI water and dried with nitrogen gas for later use.

### 2.2. Preparation of Cu_2−x_S NP Composite PI

The Cu_2−x_S composite film was obtained using the Kirkendall method [52,53]. Typically, the as−prepared Cu NP composite PI film was immersed in 0.2 mmol thioureas and 20 mL ethylene glycol (20 mL) solutions. The equipment was sealed and reacted for 12 h at 80 °C. The obtained (light yellow to black−green) composite films were then rinsed with plenty of ethanol to remove the residuals and dried with nitrogen gas for use.

### 2.3. Photocatalytic Test

To assess the photocatalytic capacities of the prepared Cu_2−x_S/PI composite film, Rhodamine (RhB) was first selected as a tentative organic material. In each test, the composite film (4 × 5 cm^2^, rolling into a cylindrical ring) was immersed in the glass bottle with 15 mL of RhB aqueous solution, and then, 200 µL of hydrogen peroxide (30 wt%) was added. Sequentially, 2 mL of the RhB solution was extracted every 10 min after the light was irradiated. The absorbance of the extracted solution was collected using a UV−vis−NIR spectrophotometer to estimate the rate of photodegradation.

Similarly, the indoor HCHO removal capacity was preceded as follows: the synthesized composite film (2.5 × 4 cm^2^, cylindrical ring) was placed in 7 of the same little bottles (5 mL) containing 3 mg of HCHO aqueous solution. Each bottle was taken out after 10 min of irradiation (avoiding direct exposure to HCHO in samples that were extracted midway), adsorbed with a 50 ppm MBTH aqueous solution for 10 min, and subsequently reacted with 1% ammonium iron (III) sulfate dodecahydrate at 35 °C for 20 min. The absorbance of the resulting solutions was also collected. The HCHO removal was calculated using the following formula:

HCHO removal efficiency (%) = (*C*_0_ − *C*_t_)/*C*_0_ × 100 (1)

where *C*_0_ and *C*_t_ are the HCHO concentrations which refer to absorbance before and after the catalytic reaction within 60 min, respectively.

### 2.4. Characterization

The crystallinity and morphology of the Cu_2−x_S/PI films were observed via XRD (Empyrean, PANalytical B.V., Netherlands), high−resolution transmission electron microscopy (HRTEM, Talos F200X, FEI), and FE−SEM (Nova NanoSEM 450, FEI, Waltham, MA, USA). Note: To prepare the TEM samples, the PI substrate had to be dissolved away because of the robust adhesion between the Cu_2−x_S and PI, which was too strong to be obtained simply through ultrasonication within a short time. A UV−vis−NIR spectrophotometer (SolidSpec−3700, Shimadzu, Japan) was adopted to monitor the NIR−shielding efficiency. To confirm the changes during the nanostructure growth process, FT−IR (Nicolet iS50R, Thermo Scientific, Waltham, MA, USA) and XPS (AXIS Supra+, Shimadzu−Kratos, Japan) were employed. The cyclic bending test was conducted using a homemade reciprocating extensometer to explore the durability of Cu_2−x_S/PI films. After 5000 bending cycles, the NIR−shielding performance and haze factor values were collected. The light source (100 mW∙cm^−2^) was utilized via a 300 W xenon lamp (Newport, model 69911, Boston, MA, USA) coupled with an AM 1.5 G filter.

## 3. Results and Discussion

### 3.1. In Situ Growth of Cu_2−x_S NPs on PI Films

**Figure 1:**
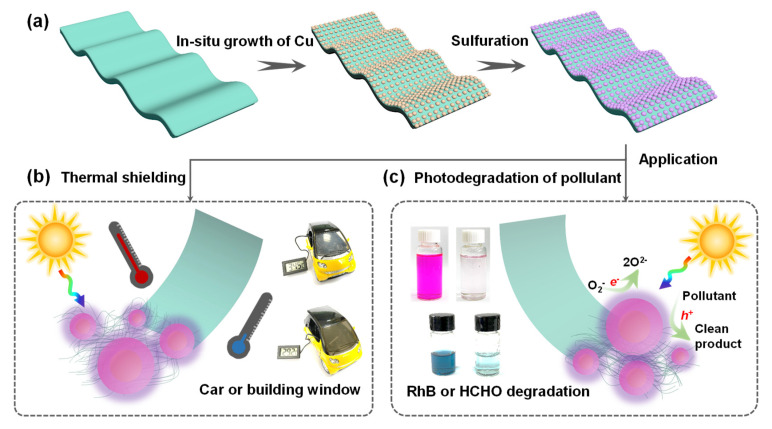
Schematic illustration of (**a**) Cu_2−x_S NPs’ in situ growth on the colorless polyimide (PI) film and their corresponding application in (**b**) thermal−shielding composite film integrated to model car windows, (**c**) photocatalytic film in degradation of RhB and HCHO.

**Figure 2:**
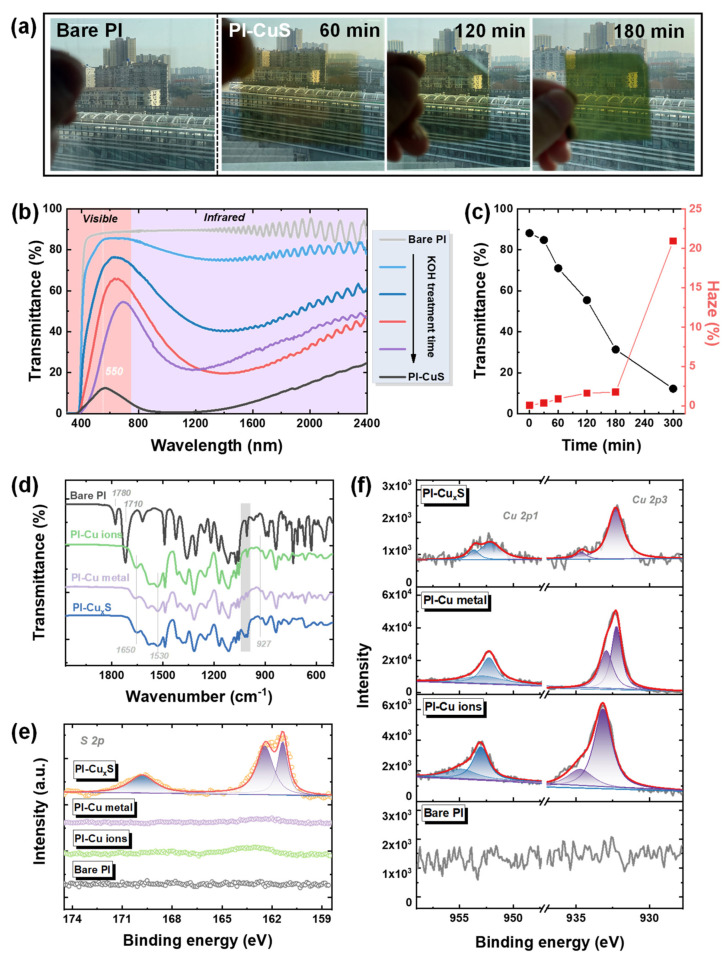
(**a**) Optical photographs through the glass window, (**b**) UV−vis−NIR, and (**c**) the collected values of transmittance at 550 nm and corresponding haze factor of Cu_2−x_S/PI composite film with respect to KOH treatment time (namely Cu_2−x_S densities). (**d**) Polymer spectrum of Cu_2−x_S growth process and corresponding XPS spectra of S 2p (**e**) and Cu 2p (**f**).

Figure 1 illustrates the processes of the facile−integrated nanomaterial–substrate strategy to manufacture a bifunctional window film. In situ growth technology, explored herein, renders the direct integration of Cu_2−x_S nanoparticles (NPs) to PI films. As presented in Figure 1a, firstly, the copper cations were exchanged into the carboxyl groups of the alkali−cleaved imide rings in the PI outer surface, as reported in previous work [51]. Then, the metallic copper was achieved via the in situ chemical reduction of Cu^2+^ with DMAB (a detailed reaction process is plotted in Scheme S1); subsequently, the Cu_2−x_S NPs were obtained via the Kirkendall−type diffusion process. As depicted in Figure S1 and Figure 2a, the colorless PI turned brown after Cu metal attachment and gradually changed to laurel−green with S replacement because of localized surface plasmon resonance (LSPR) absorption, indicating that the Cu_2−x_S availability was substituted in situ on the metallic Cu surfaces. It was observed that the increased Cu NPs (increasing with the Cu^2+^ addition, namely lengthening KOH treatment time), led to enhanced Cu_2−x_S densities, making a darker surface due to the densely configured NPs, with a tendency toward black (Figure S1). Figure 2b exhibits the optical properties of composite films from the UV, vis, and NIR regions. The increased Cu_2−x_S reduced the overall transmittance, and the NIR blocking tendency was distinct, regardless of UV filtration from PI itself. However, these were still highly transparent and clearly visible in the scenery outside through the fabricated composite films (Figure 2a). In addition, the transparency at the wavelength of 550 nm and their corresponding haze factor were measured (Figure 2c and Table 1). As the Cu_2−x_S concentration increased, the transmittances of the composite films decreased from 88.19%, 84.80%, 71.02%, 56.44%, 31.35%, to 12.22%. The haze factor, which is the ratio of diffuse to total light transmission [54], is an essential parameter for a variety of window films. The haziness increased from 0.08, to 0.34, 0.89, 1.60, 1.74, and 20.92% with increased Cu_2−x_S densities. Low haze was ascribed to all Cu_2−x_S located at the outer layer of the film; it did not fill the entire range. Low haze values (typically 2–4%) are necessary for thermal−shielding window films in buildings or vehicles to satisfy the comfort and visibility of the human eye. To estimate the heat−insulating performance of the composite films, the shielding efficiencies in visible and NIR regions were calculated using [55]:

(2) $$T_{\mathrm{vis}}=\frac{\int_{400}^{780}T(\lambda )d\lambda }{780-400}$$

(3) $$S_{\mathrm{NIR}}=100\%-\frac{\int_{780}^{2400}T(\lambda )d\lambda }{2400-780}$$

where *T*_vis_ and *S*_NIR_ are the visible light transparency and NIR shielding values, and *T*(*λ*) refers to the optical transmittance acquired from UV−vis−NIR spectrums (Figure 2b). Table 1 summarizes the *T*_vis_, *T*_550_, haze, *S*_NIR_, and *S*_UV_ values from composite films with various Cu_2−x_S densities; *T*_550_ and *S*_UV_ are the transmittances at 550 nm and shielding values in the UV region, respectively. As a result, hundred−percent UV−blocking was obtained in all Cu_2−x_S/PI composite films, and the films after 120 min of KOH treatment showed superior thermal−shielding properties from 69.45% to 91.57%, along with a transmittance decrease from 50.01% to 7.98%. Collectively, this in situ growth fabrication method with 120 min of KOH processing time was demonstrated as optimal, which yielded high transparency (56.44% at 550 nm, 66.13% at 630 nm, and 50.01% average transmittance) and a low haze factor (1.60%).

To assess the catalytic potential of the selected film, the carrier concentration was calculated. The level of free carrier density in Cu_2__−x_S NPs results from the Cu vacancies, caused by the decreasing Cu stoichiometry and presence on top of the valence band, thus leading to absorption changes. It can be seen that the absorbance spectra of the Cu_2−x_S/PI film in Figure S2 were blue−shifted to the relatively shorter wavelength with a lower Cu_2−x_S density. The LSPR absorption peak of Cu_2__−x_S NPs was located at 1238 nm after 120 min of KOH processing. As the resonance NIR absorption is correlated with the free carrier concentration, the free carrier density of Cu_2−x_S NPs could be calculated with Mie’s theory using the following equation [56]:

(4) $$\omega =\frac{1}{2\pi }\sqrt{\frac{Ne^{2}}{\varepsilon_{0}m_{e}(\varepsilon_{\infty }+2\varepsilon_{m})}}$$

Here, *ω* refers to the LSPR frequency, *N* is the free carrier density, *e* is the electron charge, *m_e_* is the free carrier effective mass, *ε*_0_ is the permittivity of free space, *ε*_∞_ is the high−frequency dielectric constant (assumed to be 9) [57], and *ε_m_* is the dielectric constant of the surrounding medium (*ε*_m_ of PI = 2.49). From Equation (4) and Figure S2, the calculated holes (vacancy defects) value *N* of Cu_2−x_S NPs was estimated to be 1.02 × 10^22^ cm^−3^, which closely matched that in previously reported work [44]. Therefore, these abundant hole concentrations mean Cu_2−x_S/PI films are promising candidates for LSPR−enhanced photocatalysts, especially in the vis−NIR region.

In turn, the NIR−shielding performance of Cu_2−x_S/PI can be estimated with the hole density value *N* by the Drude–Lorentz model, as follows [58,59]:

(5) $$\alpha =\frac{e^{2}N}{m_{e}\varepsilon_{0}nc\tau \omega_{in}^{2}}$$

where α refers to the absorption coefficient, *c* and *ω_in_* are the incident light speed and frequency, *τ* presents the mean time between two charge carrier scattering events, and *n* is the refractive index of Cu_2−x_S. Formula (4) demonstrates an exactly linear dependence between the absorption coefficient α with the hole density *N*. In this regard, the high hole concentration in Cu_2−x_S via in situ growth fabrication provides the foundation for its utilization in thermal shielding and pollutants’ photodegradation.

### 3.2. Morphology and Structure Observations of Cu_2−x_S/PI Film

**Figure 3:**
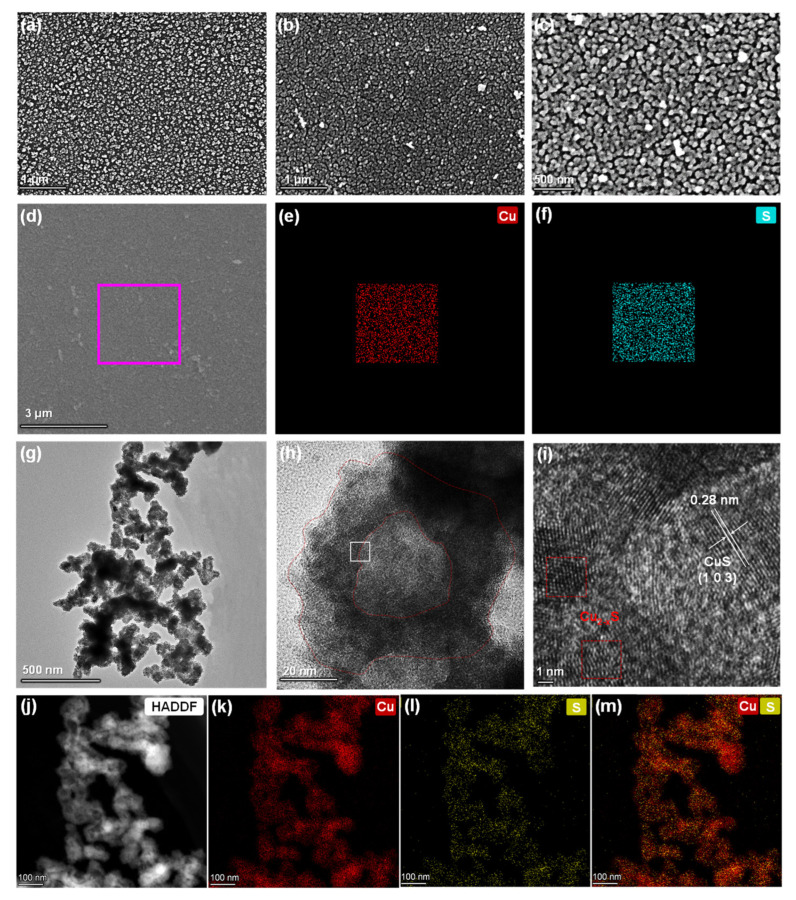
The scanning electron microscopy (SEM) images of PI film with (**a**) metallic Cu, (**b**,**c**) Cu_2−x_S NPs, and (**d**–**f**) the corresponding energy−dispersive X−ray spectrometry (EDXS) mapping results. (**g**,**h**) The transmission electron microscopy (TEM) images of Cu_2−x_S derived from PI with different magnifications, and (**i**) the corresponding lattice fringes of the selected area. (**j**–**m**) The high−angle annular dark field (HAADF) and EDXS mapping images of Cu_2−x_S NPs.

To confirm the successful growth of Cu_2−x_S, the fabricated processes were monitored using ATR−FTIR spectra, as presented in Figure 2d. The carbonyl stretching of imide rings in PI with characteristic bands at 1780 cm^−1^ (symmetric) and 1710 cm^−1^ (antisymmetric) totally disappeared after KOH treatment. New bands were observed at 1650 and 1530 cm^−1^, which were ascribed to the amide bond including carbonyl stretching and N–H bending after ring cleavage, respectively. After copper cation exchange or reduction into metallic Cu, and even sulfur diffusion to Cu_2−x_S, the spectrum was nearly the same; the mild change around 1000 cm^−1^ implied that several corresponding states were substituted for the Cu^2+^ and Cu complexes. X−ray photoelectron spectroscopy (XPS) analysis was employed to monitor the surface change during the Cu_2−x_S growth process. As plotted in Figure S3, strong Cu 2p peaks (950.5 and 931.2 eV) and Cu 3p peaks (73.5 eV) were observed after chemical reduction, revealing the presence of Cu NPs on the surface, in comparison to bare PI film. Additionally, after S diffusion, the S 2p peaks (169.9 and 161.9 eV) in Figure 2e gradually arose with Cu 2p, and the N 1s peak comparatively decreased rather than completely disappeared (Figure 2f and Figure S4), which proves the Cu_2−x_S nanostructure was probably formed. Scanning electron microscope (SEM) measurement was utilized to confirm the morphology and distribution of the NPs, as shown in Figure 3 and the size histogram in Figure S5. The uniform distribution of Cu NPs with a diameter of 58.5 ± 0.25 nm attached to the PI surface after chemical reduction is shown in Figure 3a. Additionally, there exists a slightly increased size value (59.5 ± 0.15 nm) after sulphuration in Figure 3b,c, showing that Cu_2−x_S NPs were uniformly covered on the PI surface, and negligible change in morphology was observed after S^2−^ diffusion. It was observed that the uniformly distributed Cu_2−x_S NPs on film undoubtedly facilitated the NIR absorption without the aggregation of particles. The corresponding SEM energy−dispersive spectrum (SEM−EDS) mapping of the composite film (Figure 3d–f) demonstrates an obvious S layer (cyan dots in Figure 3f) evenly covered the Cu layer (red dots in Figure 3e) after sulphuration, and the atomic ratio of Cu to S was calculated to 1.85:1 in Figure S6. Additionally, to explore the Cu_2−x_S NPs’ structure, a transmission electron microscope (TEM) was used. As plotted in Figure 3g–i, the hollow structures of Cu_2−x_S were 10–20 nm in thickness, and the average size slightly increased to 62.3 ± 1.20 nm (Figure S5c), and from this confirmation, the particles’ size and shape were considered uniform. The high−resolution transmission electron microscopy (HRTEM) image in Figure 3i, a selected region at the hollow boundaries (inner) of the NPs (Figure 3h), shows one well−resolved lattice fringe with a spacing of 0.28 nm, which corresponds to the CuS (103) plane interplanar distance. This plane direction is consistent with the major peak of the X−ray diffractometry (XRD) patterns (Figure S7, JCPDS reference: 06−0463). However, in the other lattice fringes (red box region in Figure 3i) and peaks at 33.6 and 37.5 degrees in XRD patterns, a perfectly matched preference for the formation, which is referred to as Cu_2−x_S in the literature, could not be observed [60]. Moreover, the high−angle annular dark−field (HAADF) STEM and energy−dispersive X−ray spectroscopy (EDX) maps (Figure 3j–m) of Cu_2−x_S NPs indicated a clear distribution of two elements (Cu and S), with significantly increased S elements located on the outer surface of NPs, which strongly confirmed the existence of hollow structures. It is speculated that the Kirkendall−type diffusion process [52] could explain this hollowing mechanism of Cu_2−x_S NPs. The CuS thin layer was firstly nucleated and formed on the outer surface of Cu seeds, which then acted as an interface and barrier with the inner Cu cations and outside S^2−^ ions in the bulk solution, hindering the direct reaction of Cu with S^2−^. Then, atomic diffusion occurred through vacancy exchange, thus forming the hollow Cu_2−x_S structures as previously reported [61]. Encouragingly, hollow nanostructures can offer numerous reaction sites and a larger surface area, which facilitates the reacted ratio during photocatalysis, and the collection and utilization of solar irradiation are also enhanced through light scattering and reflection, thus optimizing the catalytic efficiency.

### 3.3. Thermal−Shielding Performance Evaluation

**Figure 4:**
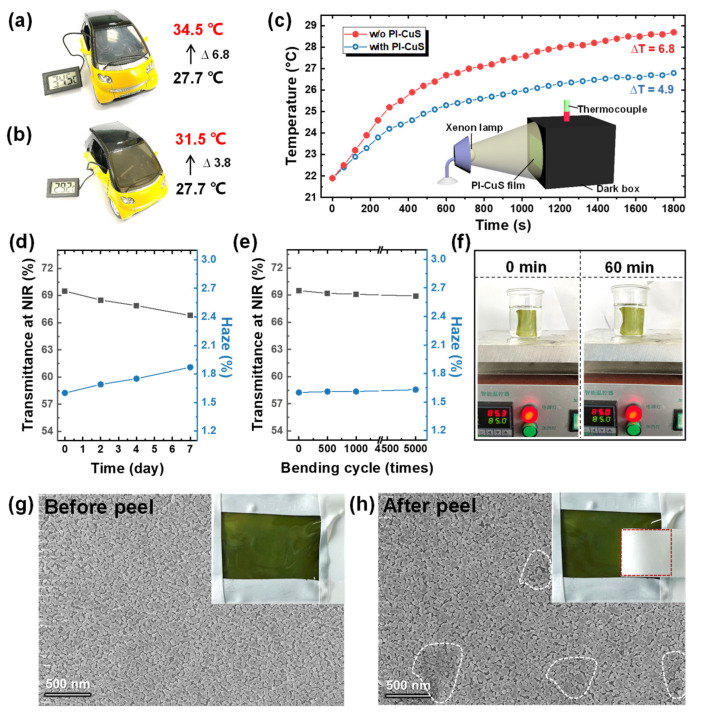
The investigation of the temperature changes in the model car (**a**) without and (**b**) with attaching Cu_2−x_S/PI film on the windows under real sun irradiation. (**c**) Monitoring the interior temperature changes in the sealed box with quartz glass and glass/PI−Cu_2−x_S attached to a facet as a function of exposure time. (Inset: schematic illustration of the simulated experiment exposure with xenon lamp irradiation with an intensity of 100 mW∙cm^−2^ onto a facet of the sealed box). Monitoring NIR shielding performance (gray) and haze factor (blue) variation of Cu_2−x_S/PI films under (**d**) high temperature and high humidity (85 °C/85%RH) test for 7 days, and (**e**) cyclic bending 5000 times. (**f**) Cu_2−x_S/PI films were soaked in water for 1 h at 85 °C (no obvious change observed). SEM images of the Cu_2−x_S/PI film before (**g**) and after (**h**) peel tests with adhesive tape (inset: no detectable Cu_2−x_S stripped off by tape (red region)).

To evaluate the thermal−shielding capability of Cu_2−x_S/PI composites films, Figure 4 exhibits the investigation of temperature variation on composite films directly attached to the window of the model car and simulated building. Regarding the model car (1:16 scale), a comparative test of thermal shielding between the presence and absence of Cu_2−x_S/PI films onto the car window is shown in Figure 4a,b. Primitively, the temperature in the car was 27.7 °C and then increased to 34.5 and 31.5 °C for the car with no film (Figure 4a), and with the Cu_2−x_S/PI film (Figure 4b), respectively, after exposure to direct sunlight irradiation for 1 h. This result reveals that composite films can block sunlight. Meanwhile, the simulation thermal shielding in a building was performed using irradiation with and without an attached Cu_2−x_S/PI film using a xenon lamp (simulated solar irradiation). In this test, the composite films and glass acted as window films, and the window was attached to the sealed dark acrylic box, as depicted in Figure 4c inset. The temperature evolution as a function of exposure time was collected. Before irradiation, the initial temperature was 21.9 °C. Figure 4c plots the results after irradiation for 1800 s. The interior temperature with bare glass rapidly increased to 28.7 °C (ΔT = 6.8 °C), whereas the temperature with the Cu_2−x_S/PI film attached slowly increased to 26.8 °C (ΔT = 4.9 °C). These results effectively demonstrate the potential of the composite films to perform heat−shielding functions to save energy. To determine their potential application as a window film, the reliability test for Cu_2−x_S/PI films was conducted by investigating transmittance and haze changes in extreme environments (85 °C temperature and 85% humidity). During the 7−day−long tests, no obvious change was monitored in the NIR−shielding performance and haze factor (Figure 4d and Figure S8). Additionally, the Cu_2−x_S/PI composite films possessed consistent efficiency under cyclic mechanical bending up to 5000 times, as shown in Figure 4e. The collected NIR absorption and haze values also exhibited no significant change during the bending test, shown in Figure S9 (180° bending with a curvature radius of 6 mm). Thus, this in situ fabrication of Cu_2−x_S/PI composite films demonstrated the highly effective and reliable thermal−shielding capability of the film.

### 3.4. HCHO Removal Using Cu_2−x_S/PI Film

**Figure 5:**
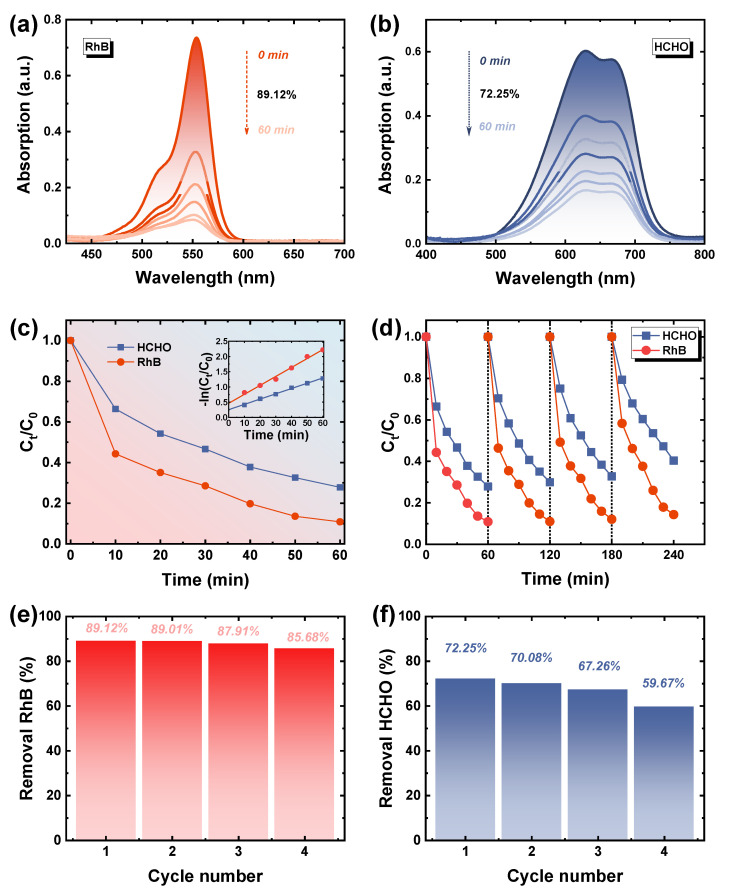
The absorption spectra of the photocatalytic degradation of (**a**) RhB and (**b**) HCHO solution (MBTH method) in the presence of Cu_2−x_S/PI films under Vis−NIR light irradiation for 60 min, and (**c**) the corresponding degradation rate and kinetic curves (inset). (**d**) Cycling test for the photodegradation of RhB (red) and HCHO (blue) under their corresponding degeneration rate of (**e**) RhB and (**f**) HCHO solution.

We carried out the assessment of the possibility of Cu_2−x_S/PI composite films to be utilized as a dual−function window films. The adhesion strength between surface Cu_2−x_S and PI should be guaranteed. As demonstrated in Figure 4f and Video S1, no detectable change was observed when the composite film was placed in the heated solution (85 °C) for 1 h. An adhesive peel test was also conducted (see Videos S2 and S3), and after being pasted and peeled with strong adhesive tape, the composite film exhibited no significant change, strongly indicating the robust adhesion between surface Cu_2−x_S and PI. The SEM results (Figure 4g,h) also proved that, although a small amount of Cu_2−x_S blur (white area) existed after peeling, the overall regional particles were fully retained, which provide a guarantee for reaching catalytic stability. The catalytic capacities for organic pollutant removal were evaluated. The rate of Rhodamine (RhB) and HCHO catalytic oxidation with Cu_2−x_S/PI was measured (details in experimental section and Figure S10) at room temperature. Figure 5a,b are the spectrums of RhB and HCHO (adsorbed with MBTH and reacted with ammonium ferric sulfate) solution absorbance changing with reaction time; their degradation rate was calculated by comparing the absorption change at 554 and 630 nm. Consequently, benefitting from the dense NPs with hollow structures, the removal rates of RhB and HCHO for Cu_2−x_S/PI reached 89.12% and 72.25%, respectively, within 60 min. The degradation rate was fastest within the initial 10 min, and the reaction was ultimately completed within 150 min in RhB (99.0%) and 240 min in HCHO (98.7%), as shown in Figures S11 and S12, respectively. The difference between the two mainly originated from the diverse size of composite films used (Figure S10) because of the reactor limitation. According to Figure 5c, Figures S11b and S12 inset, the RhB and HCHO degradation via the Cu_2−x_S/PI composite films demonstrated in the first−order reaction kinetics model (–ln(C_t_/C_0_) = *k*t), and the kinetic constant (*k*) in RhB and HCHO was calculated to be 0.0292 and 0.0174 min^−1^. Expectedly, during the cycle test, the decline in catalytic efficiency was inevitable after catalyst re−collection and the washing process, whereas the robust adhesion between Cu_2−x_S and PI, without any collection, enabled the degradation rate of RhB to still reach 85% and nearly 60% in HCHO in 60 min after four cycles, which maintained a stable removal efficiency of 96.1% in RhB and 82.6% in HCHO with the composite films, as plotted in Figure 5d–f. In addition, the Cu_2−x_S/PI exhibited structural and chemical stability after four cycles, as evidenced by the results of the XRD analysis; the film had no obvious changes and still maintained good crystallinity (Figure S13) after four cycles, demonstrating high reusability. Expectedly, the stability of Cu_2−x_S/PI was inferior to the material produced in the commonly used dispersion coating method, in which particles are entirely distributed inside the film. For future industrial usage, we can keep the Cu_2−x_S/PI composite films layer by layer with optical clear adhesive, as shown in Figure S14; the upper layer coverage can efficiently prevent the Cu_2−x_S on the next layer from deteriorating because they need not be exposed to the ambient environment. Additionally, robust adhesion provides the Cu_2−x_S with complete preservation when the upper layer is subsequently peeled off for use, which can greatly extend the service and storage life of the composite film.

## 4. Conclusions

In summary, the hollow Cu_2−x_S was successfully integrated into PI films through facile in situ growth with high stability and homogeneity, which were applied as thermal−shielding and HCHO photodegradation films for windows. The experimental results show that the Cu_2−x_S/PI composite film had consistent efficiency in NIR filtration and the haze factor after the reliability (85 °C−85%RH) test as well as after 5000 cyclic bending cycles. The composite films with optimal densities of Cu_2−x_S demonstrated superior NIR−shielding efficiency (69.4%) and excellent heat−blocking when directly attached to the window of a model car and simulated building. Moreover, with the distinctive distribution and hollow structure of Cu_2−x_S, this enabled the successful utilization of the additional thermal energy from NIR harvesting and provided good HCHO removal efficiency (72%) within 60 min and high reusability after four cycles. These findings can provide a new method for the generation of thermal−shielding and indoor organic−pollutant−removal bifunctional window films.

## Figures and Tables

**Table 1:** Transmittance and absorption values in NIR region, calculated from Figure 2b.

	Treatment Time (min)	0	30	60	120	180	300
	
Transmittance (@ 550 nm, %)		88.19	84.80	71.02	56.44	31.35	12.22
Average Transmittance (400–780 nm, %)		86.99	81.27	65.31	50.01	35.08	7.98
Haze (%)		0.08	0.34	0.89	1.60	1.74	20.92
Average shielding rate in the NIR region (780–2400 nm)		10.43	21.63	49.94	69.45	66.40	91.57
Average shielding rate in the UV region (300–400 nm)		100	100	100	100	100	100

## Data Availability

The data presented in this study are available on request from the corresponding author.

## Supplementary Materials

The following supporting information (Description of the material, figures as described in the text included) can be downloaded at: https://www.mdpi.com/article/10.3390/polym14163382/s1. Scheme S1: Schematic of the Cu nanostructure formation; Figure S1: Optical photographs of PI film covered metal cooper and Cu_2−x_S with respect to KOH treatment time; Figure S2: The absorbance spectra of the PI films with the various density of Cu_2−x_S NPs; Figure S3: Survey X−ray photoelectron spectra of Cu_2−x_S growth on the PI process; Figure S4: XPS spectra of N 1s in Cu_2−x_S growth process; Figure S5: Size histograms of Cu and Cu_2−x_S NPs from Figure 3; Figure S6: Element quantitative analysis of Cu_2−x_S on PI from Figure 3; Figure S7: XRD patterns change during the Cu_2−x_S growth process; Figure S8: Typical photographs of the Cu_2−x_S/PI film before and after the high−temperature and high−humidity test (85 °C/85%RH test) for 7 days; Figure S9: Bending test of PI−Cu_2−x_S film loaded on the bending test machine; Figure S10: Photocatalytic test; Figure S11: The absorption spectra of the photocatalytic degradation and the corresponding degradation rate and kinetic curves (inset)of RhB solution in the presence of PI−Cu_2−x_S films; Figure S12. The absorption spectra of the photocatalytic degradation corresponding degradation rate and kinetic curves (inset) of HCHO solution in the presence of PI−Cu_2−x_S films (2.5 × 4 cm^2^); Figure S13: XRD comparison diagram between original and after 4 cycles of testing samples; Figure S14. Schematic illustration of improved service life of composite films by the layer by layer method; Video S1: The composite film placed in the heated solution (85 °C) for 1 h; Videos S2 and S3: The adhesive peel test.

## References

[1] Vahidi A., Sciarretta A. (2018). Energy saving potentials of connected and automated vehicles. Transport. Res. C−Emerg..

[2] Asefi G., Habibollahzade A., Ma T., Houshfar E., Wang R. (2021). Thermal management of building−integrated photovoltaic/thermal systems: A comprehensive review. Sol. Energy.

[3] Aburas M., Soebarto V., Williamson T., Liang R., Ebendorff−Heidepriem H., Wu Y. (2019). Thermochromic smart window technologies for building application: A review. Appl. Energy.

[4] Smalyukh I.I. (2021). Thermal management by engineering the alignment of nanocellulose. Adv. Mater..

[5] Gao H., Koch C., Wu Y. (2019). Building information modelling based building energy modelling: A review. Appl. Energy.

[6] Park K., Jin S., Kim G. (2021). Transparent window film with embedded nano−shades for thermoregulation. Consstr. Build. Mater..

[7] Lambin P., Liubimau A., Bychanok D., Vitale L., Kuzhir P. (2020). Thermal and electromagnetic properties of polymer holey structures produced by additive manufacturing. Polymers.

[8] Song M., Jiang J., Qin H., Ren X., Jiang F. (2020). Flexible and super thermal insulating cellulose nanofibril/emulsion composite aerogel with quasi−closed pores. ACS Appl. Mater. Interfaces.

[9] Connelly K., Wu Y., Chen J., Lei Y. (2016). Design and development of a reflective membrane for a novel Building Integrated Concentrating Photovoltaic (BICPV) ‘Smart Window’ system. Appl. Energy.

[10] Center R.R.D. (2009). Reference Solar Spectral Irradiance: ASTM G−173.

[11] Xiang B., Zhang J. (2018). A new member of solar heat−reflective pigments: BaTiO_3_ and its effect on the cooling properties of ASA (acrylonitrile−styrene−acrylate copolymer). Sol. Energy Mater. Sol. Cells.

[12] Qi Y., Xiang B., Zhang J. (2017). Effect of titanium dioxide (TiO_2_) with different crystal forms and surface modifications on cooling property and surface wettability of cool roofing materials. Sol. Energy Mater. Sol. Cells.

[13] Ding C., Han A., Ye M., Zhang Y., Yao L., Yang J. (2019). Synthesis and characterization of a series of new green solar heat−reflective pigments: Cr−doped BiPO_4_ and its effect on the aging resistance of PMMA (Poly (methyl methacrylate)). Sol. Energy Mater. Sol. Cells.

[14] Radhika S.P., Sreeram K.J., Unni Nair B. (2014). Mo−doped cerium gadolinium oxide as environmentally sustainable yellow pigments. ACS Sustain. Chem. Eng..

[15] Liu J., Zhang J., Tang H., Zhou Z., Zhang D., Ye L., Zhao D. (2021). Recent advances in the development of radiative sky cooling inspired from solar thermal harvesting. Nano Energy.

[16] Wang M., Xu Y., Liu Y., Wu W., Xu S. (2019). Synthesis of Sb−doped SnO_2_ (ATO) hollow microspheres and its application in photo−thermal shielding coating. Prog. Org. Coat..

[17] Khanyile B., Madiba I., Mtshali C., Mabakachaba B., Moloi S., Nkosi M., Maaza M. (2022). Effect of the bottom layer thickness on the structural and optical phase transition properties of V_2_O_5_/V/V_2_O_5_ thin films. Mater. Today.

[18] Wen R.−T., Granqvist C.G., Niklasson G.A. (2015). Eliminating degradation and uncovering ion−trapping dynamics in electrochromic WO_3_ thin films. Nat. Mater..

[19] Yang J., Xu Z., Ye H., Xu X., Wu X., Wang J. (2015). Performance analyses of building energy on phase transition processes of VO_2_ windows with an improved model. Appl. Energy.

[20] Sun H., Xie Z., Ju C., Hu X., Yuan D., Zhao W., Shui L., Zhou G. (2019). Dye−doped electrically smart windows based on polymer−stabilized liquid crystal. Polymers.

[21] Sousa−Castillo A., Ameneiro−Prieto Ó., Comesaña−Hermo M., Yu R., Vila−Fungueiriño J.M., Pérez−Lorenzo M., Rivadulla F., García de Abajo F.J., Correa−Duarte M.A. (2017). Hybrid plasmonic nanoresonators as efficient solar heat shields. Nano Energy.

[22] Yang S.−G., Liu J.−C., Yang Y., Fang Q., Rong C., Gan J.−P. (2020). Experimental investigation of rupture and dispersion characteristics of float glass subjected to vented explosion loads of methane−air mixtures. Int. J. Impact Eng..

[23] Longcore T., Rich C. (2004). Ecological light pollution. Front. Ecol. Environ..

[24] Harima T., Nagahama T. (2017). Evaluation methods for retroreflectors and quantitative analysis of near−infrared upward reflective solar control window film—Part I: Theory and evaluation methods. Sol. Energy.

[25] Chao L., Bao L., Wei W., Tegus O. (2019). A review of recent advances in synthesis, characterization and NIR shielding property of nanocrystalline rare−earth hexaborides and tungsten bronzes. Sol. Energy.

[26] He C., Cheng J., Zhang X., Douthwaite M., Pattisson S., Hao Z. (2019). Recent advances in the catalytic oxidation of volatile organic compounds: A review based on pollutant sorts and sources. Chem. Rev..

[27] Silas K., Ghani W.A.W.A.K., Choong T.S., Rashid U. (2019). Carbonaceous materials modified catalysts for simultaneous SO_2_/NO_x_ removal from flue gas: A review. Catal. Rev..

[28] Vikrant K., Kim K.−H., Deep A. (2019). Photocatalytic mineralization of hydrogen sulfide as a dual−phase technique for hydrogen production and environmental remediation. Appl. Catal. B−Environ..

[29] Tang X., Bai Y., Duong A., Smith M.T., Li L., Zhang L. (2009). Formaldehyde in China: Production, consumption, exposure levels, and health effects. Environ. Int..

[30] Dai Z., Yu J., Si Y. (2022). Gradient porous structured MnO_2_−nonwoven composite: A binder−free polymeric air filter for effective room−temperature formaldehyde removal. Polymers.

[31] Ye J., Zhou M., Le Y., Cheng B., Yu J. (2020). Three−dimensional carbon foam supported MnO_2_/Pt for rapid capture and catalytic oxidation of formaldehyde at room temperature. Appl. Catal. B−Environ..

[32] Hu F., Peng Y., Chen J., Liu S., Song H., Li J. (2019). Low content of CoOx supported on nanocrystalline CeO_2_ for toluene combustion: The importance of interfaces between active sites and supports. Appl. Catal. B−Environ..

[33] Huang Y., Long B., Tang M., Rui Z., Balogun M.−S., Tong Y., Ji H. (2016). Bifunctional catalytic material: An ultrastable and high−performance surface defect CeO_2_ nanosheets for formaldehyde thermal oxidation and photocatalytic oxidation. Appl. Catal. B−Environ..

[34] Wang Z., Wang W., Zhang L., Jiang D. (2016). Surface oxygen vacancies on Co_3_O_4_ mediated catalytic formaldehyde oxidation at room temperature. Catal. Sci. Technol..

[35] Ye J., Cheng B., Yu J., Ho W., Wageh S., Al−Ghamdi A.A. (2022). Hierarchical Co_3_O_4_−NiO hollow dodecahedron−supported Pt for room−temperature catalytic formaldehyde decomposition. Chem. Eng. J..

[36] Wang C., Li Y., Zhang C., Chen X., Liu C., Weng W., Shan W., He H. (2021). A simple strategy to improve Pd dispersion and enhance Pd/TiO_2_ catalytic activity for formaldehyde oxidation: The roles of surface defects. Appl. Catal. B−Environ..

[37] Zhang S., Zhuo Y., Ezugwu C.I., Wang C.−C., Li C., Liu S. (2021). Synergetic molecular oxygen activation and catalytic oxidation of formaldehyde over defective MIL−88B (Fe) nanorods at room temperature. Environ. Sci. Technol..

[38] Liu M., Liu Y., Gu B., Wei X., Xu G., Wang X., Swihart M.T., Yong K.−T. (2019). Recent advances in copper sulphide−based nanoheterostructures. Chem. Soc. Rev..

[39] Coughlan C., Ibanez M., Dobrozhan O., Singh A., Cabot A., Ryan K.M. (2017). Compound copper chalcogenide nanocrystals. Chem. Rev..

[40] Liu X., Swihart M.T. (2014). Heavily−doped colloidal semiconductor and metal oxide nanocrystals: An emerging new class of plasmonic nanomaterials. Chem. Soc. Rev..

[41] Fenton J.L., Steimle B.C., Schaak R.E. (2018). Exploiting crystallographic regioselectivity to engineer asymmetric three−component colloidal nanoparticle isomers using partial cation exchange reactions. J. Am. Chem. Soc..

[42] Gao Q., Wu X., Huang T. (2021). Novel energy efficient window coatings based on in doped CuS nanocrystals with enhanced NIR shielding performance. Sol. Energy.

[43] Kwon Y.−T., Ryu S.H., Shin J.W., Yeo W.−H., Choa Y.−H. (2019). Electrospun CuS/PVP nanowires and superior near−infrared filtration efficiency for thermal shielding applications. ACS Appl. Mater. Interfaces.

[44] Kwon Y.−T., Lim G.−D., Kim S., Ryu S.H., Hwang T.−Y., Park K.−R., Choa Y.−H. (2018). Near−infrared absorbance properties of Cu_2− x_S/SiO_2_ nanoparticles and their PDMS−based composites. J. Mater. Chem. C.

[45] Leng C., Zhang X., Xu F., Yuan Y., Pei H., Sun Z., Li L., Bao Z. (2018). Engineering gold nanorod–copper sulfide heterostructures with enhanced photothermal conversion efficiency and photostability. Small.

[46] Xiong Y., Luo B., Chen G., Cai J., Jiang Q., Gu B., Wang X. (2019). CuS@Corn stalk/chitin composite hydrogel for photodegradation and antibacterial. Polymers.

[47] Chen Y., Zhao S., Wang X., Peng Q., Lin R., Wang Y., Shen R., Cao X., Zhang L., Zhou G. (2016). Synergetic integration of Cu_1.94_S–Zn_x_Cd_1–x_S heteronanorods for enhanced visible−light−driven photocatalytic hydrogen production. J. Am. Chem. Soc..

[48] Lai C., Zhang M., Li B., Huang D., Zeng G., Qin L., Liu X., Yi H., Cheng M., Li L. (2019). Fabrication of CuS/BiVO_4_ (0 4 0) binary heterojunction photocatalysts with enhanced photocatalytic activity for Ciprofloxacin degradation and mechanism insight. Chem. Eng. J..

[49] Xin X., Song Y., Guo S., Zhang Y., Wang B., Yu J., Li X. (2020). In−situ growth of high−content 1T phase MoS_2_ confined in the CuS nanoframe for efficient photocatalytic hydrogen evolution. Appl. Catal. B−Environ..

[50] Liaw D.−J., Wang K.−L., Huang Y.−C., Lee K.−R., Lai J.−Y., Ha C.−S. (2012). Advanced polyimide materials: Syntheses, physical properties and applications. Prog. Polym. Sci..

[51] Liu X., Hu L., Wang R., Li J., Gu H., Liu S., Zhou Y., Tu G. (2019). Flexible perovskite solar cells via surface−confined silver nanoparticles on transparent polyimide substrates. Polymers.

[52] Yin Y., Rioux R.M., Erdonmez C.K., Hughes S., Somorjai G.A., Alivisatos A.P. (2004). Formation of hollow nanocrystals through the nanoscale Kirkendall effect. Science.

[53] Wu C., Yu S.−H., Chen S., Liu G., Liu B. (2006). Large scale synthesis of uniform CuS nanotubes in ethylene glycol by a sacrificial templating method under mild conditions. J. Mater. Chem..

[54] Clausen J., Christiansen A.B., Garnaes J., Mortensen N.A., Kristensen A. (2012). Color effects from scattering on random surface structures in dielectrics. Opt. Express.

[55] Ran S., Liu J., Shi F., Fan C., Chen B., Zhang H., Yu L., Liu S.−H. (2018). Greatly improved heat−shielding performance of K_x_WO_3_ by trace Pt doping for energy−saving window glass applications. Sol. Energy Mater. Sol. Cells.

[56] Yibi Y., Chen J., Xue J., Song J., Zeng H. (2017). Enhancement of adjustable localized surface plasmon resonance in ZnO nanocrystals via a dual doping approach. Sci. Bull..

[57] Kim M.R., Hafez H.A., Chai X., Besteiro L.V., Tan L., Ozaki T., Govorov A.O., Izquierdo R., Ma D. (2016). Covellite CuS nanocrystals: Realizing rapid microwave−assisted synthesis in air and unravelling the disappearance of their plasmon resonance after coupling with carbon nanotubes. Nanoscale.

[58] Comin A., Manna L. (2014). New materials for tunable plasmonic colloidal nanocrystals. Chem. Soc. Rev..

[59] Ghosh S., Saha M., De S.K. (2014). Tunable surface plasmon resonance and enhanced electrical conductivity of in doped ZnO colloidal nanocrystals. Nanoscale.

[60] Maiti P.S., Ganai A.K., Bar−Ziv R., Enyashin A.N., Houben L., Bar Sadan M. (2018). Cu_2–x_S–MoS_2_ nano−octahedra at the atomic scale: Using a template to activate the basal plane of MoS_2_ for hydrogen production. Chem. Mater..

[61] Cao H., Qian X., Wang C., Ma X., Yin J., Zhu Z. (2005). High symmetric 18−facet polyhedron nanocrystals of Cu_7_S_4_ with a hollow nanocage. J. Am. Chem. Soc..

