# Supplementary material for: Advanced Dual−Function Hollow Copper−Sulfide−Based Polyimide Composite Window Film Combining Near−Infrared Thermal Shielding and Organic Pollutants’ Photodegradation
Source: Polymers (Basel). 2022 Aug 18;14(16):3382. doi: 10.3390/polym14163382 (PMC9413264; doi:10.3390/polym14163382)
Supplement: Supplementary file 1 [file polymers-14-03382-s001.zip › Supplementary material.pdf]

Supplementary Materials

Advanced Dual-Function Hollow Copper Sulfide-Based Polyimide Composite Window Film Combining Near-Infrared Thermal Shielding and Organic Pollutants Photodegradation

Xiangfu Liu, Jinming Ma, Jiulin Shen, Jianqiao Zhao, Chengxu Lu and Guoli Tu*

Wuhan National Laboratory for Optoelectronics, Huazhong University of Science and Technology, 1037 Luoyu Road, Wuhan 430074, China

* Correspondence: tgl@hust.edu.cn

Materials:

Potassium hydroxide (KOH), cupric nitrate ($Cu(NO_3)_2$), dimethylamino borane (DMAB), thiourea, Rhodamine (RhB), formaldehyde (HCHO), 3-methyl-1,3-benzothiazol-2(3H)-one hydrazone (MBTH), and ammonium iron (III) sulfate dodecahydrate were purchased from Sigma-Aldrich and used directly. The precursor polyamic acid solution was supplied by Wuhan Imide Optoelectronic Material Technology Co., Ltd and coated to colorless PI film after thermal imidization by ourselves.

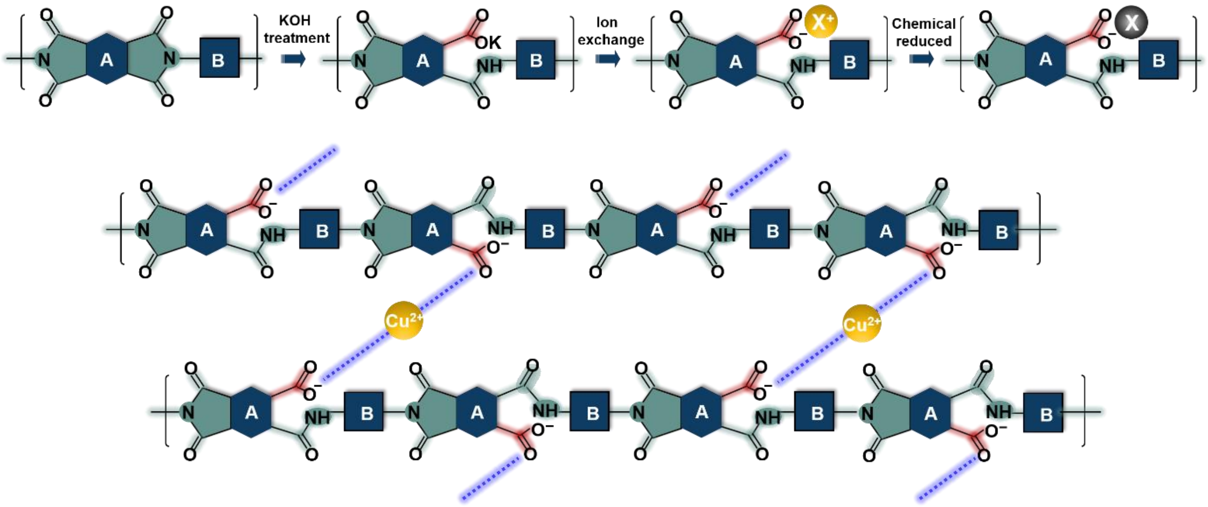

Scheme 1. Schematic of the induced metallic Cu on polyimide film, including imide ring–opening, Cu ion exchange, and Cu nanostructure formation through chemical reduction.

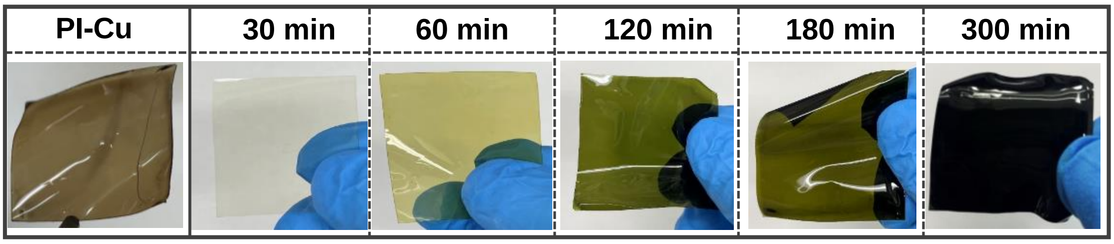

Figure S1. Optical photographs of PI film coverd metal cooper and then sulfureted to $Cu_{2-x}S$ with respect to KOH treatment time (namely particle densities).

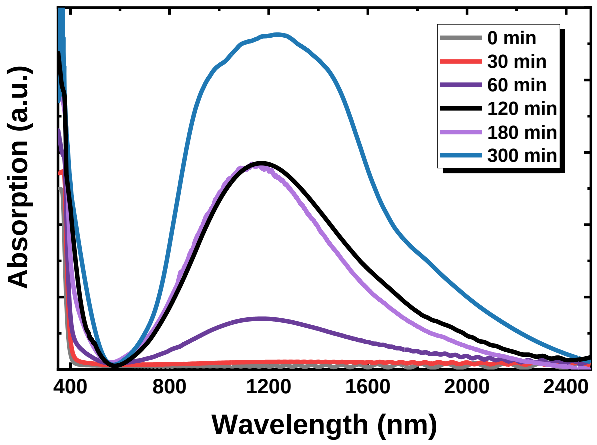

Figure S2. The absorbance spectra of the PI films with the various density of $Cu_{2-x}S$ NPs.

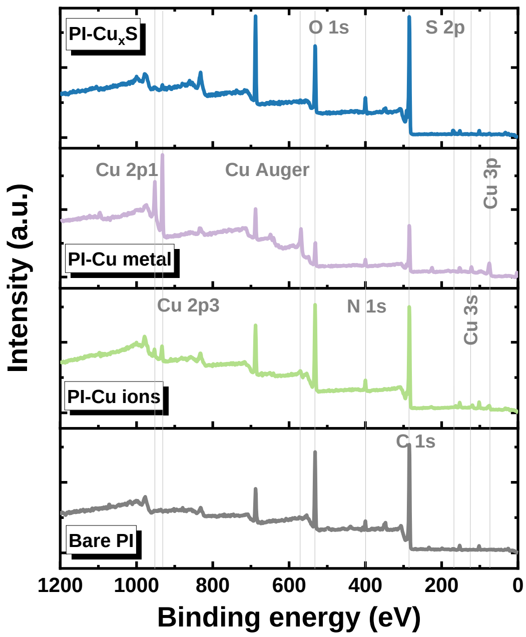

Figure S3. Survey X-ray photoelectron spectra of $Cu_{2-x}S$ growth on the PI process.

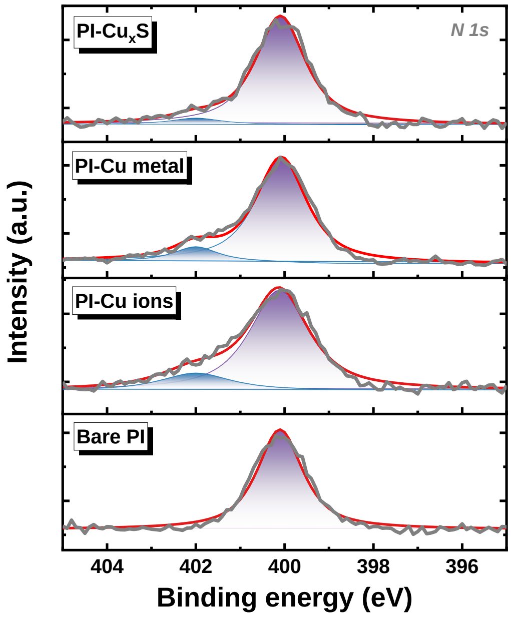

Figure S4. XPS spectra of N 1s in $Cu_{2-x}S$ growth process.

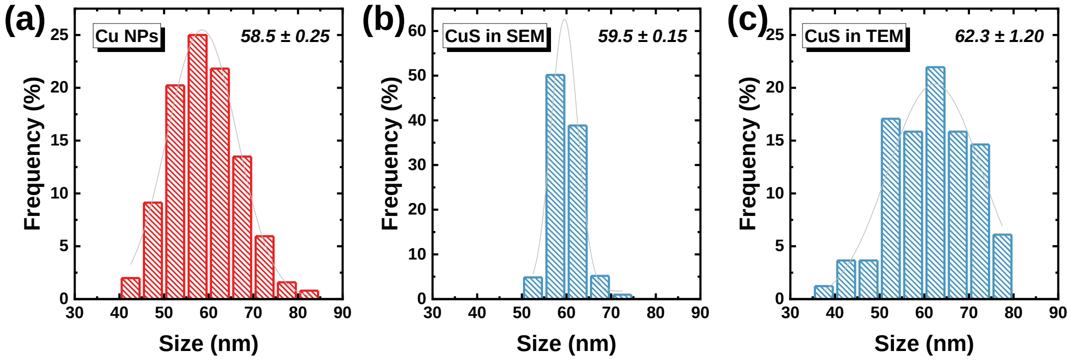

Figure S5. Size histograms of (a) Cu, $Cu_{2-x}S$ NPs in (b) SEM and (c) TEM results from **Figure 3.**

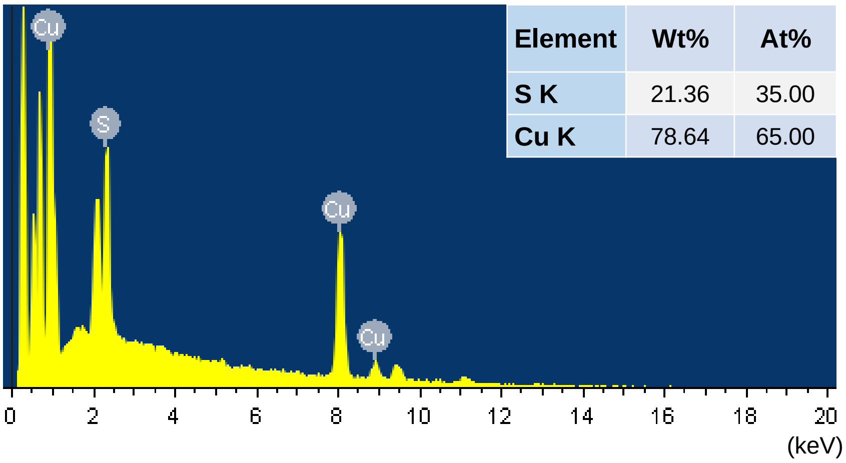

Figure S6. Element quantitative analysis of $Cu_{2-x}S$ on PI by SEM-EDXS mapping result from **Figure 3**.

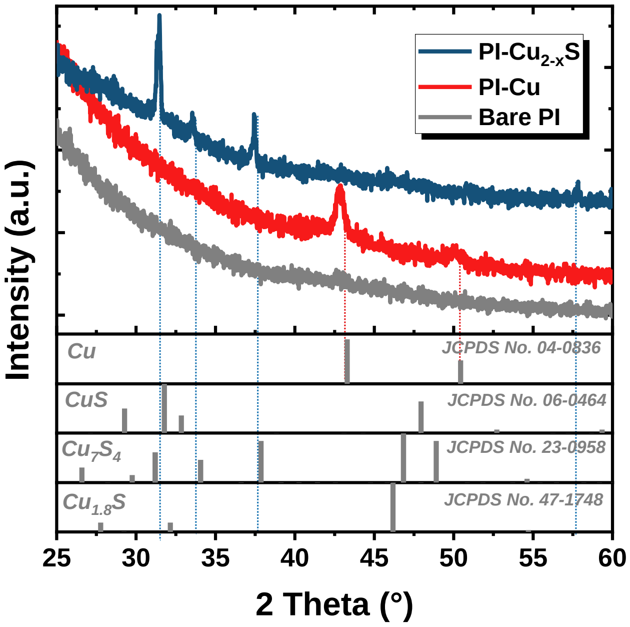

Figure S7. XRD patterns change during the $Cu_{2-x}S$ growth process.

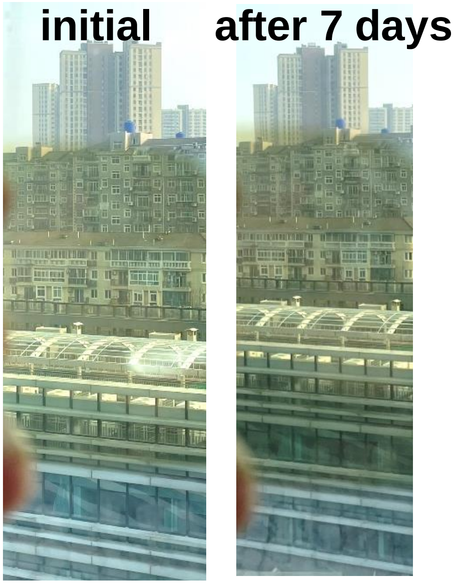

Figure S8. Typical photographs of the $Cu_{2-x}S$/PI film before and after the high-temperature and high-humidity test (85°C/85%RH test) for 7 days.

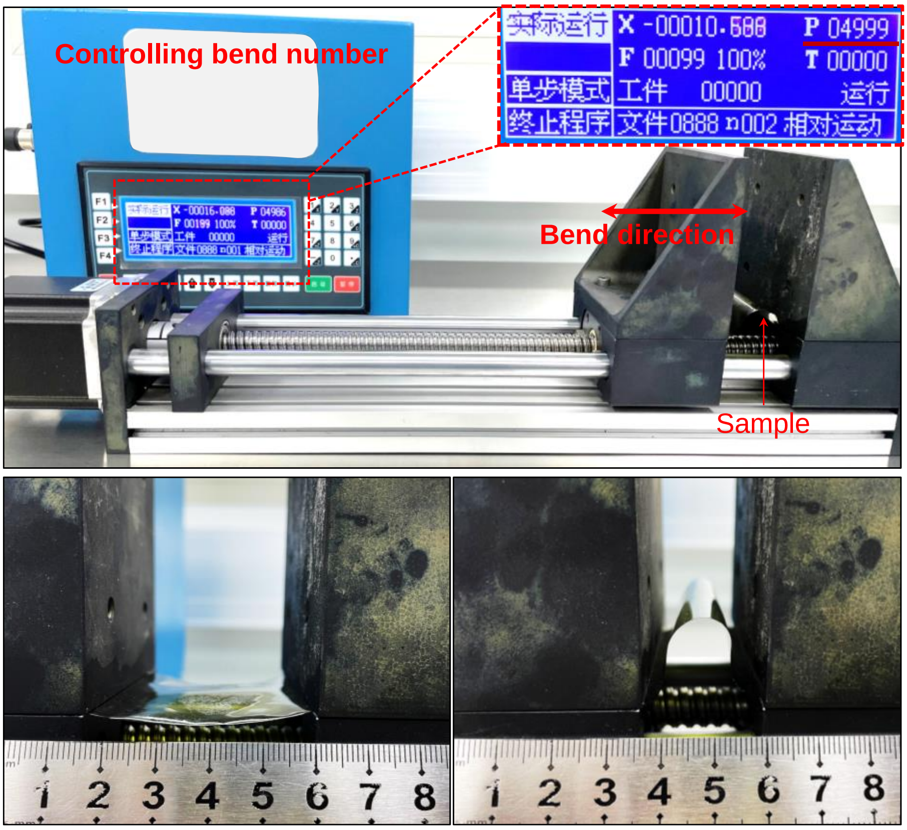

Figure S9. Bending test. The optical images of PI-CuS film were loaded on the bending test machine.

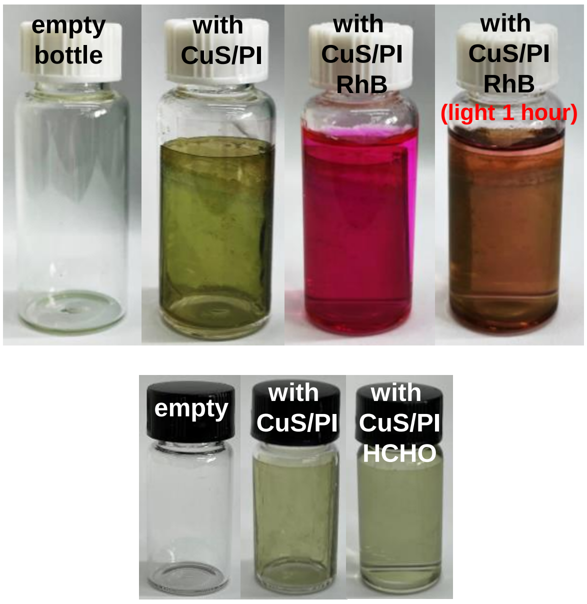

Figure S10. Photocatalytic test. the photograph for photodegradation of RhB and HCHO with thin $Cu_{2-x}S$/PI films: For RhB, the composites film (4×5 cm^2) rolling into a cylindrical ring, was immersed in the glass bottle with 15 mL RhB aqueous solution, and then 2 ml of the RhB solution was taken out every 10 min after light source irradiated. To prevent direct exposure to HCHO, the halfway extraction like in RhB is not taken. Instead, The composite film (2.5×4 cm2, roll to cylindrical ring) was placed in 7 of the same little bottle (5 mL) containing 3 mg formaldehyde aqueous solution. Take out a bottle after each 10 minutes irradiation, then adsorbed with MBTH aqueous solution and reacted with ammonium iron (III) sulfate dodecahydrate for later measurement.

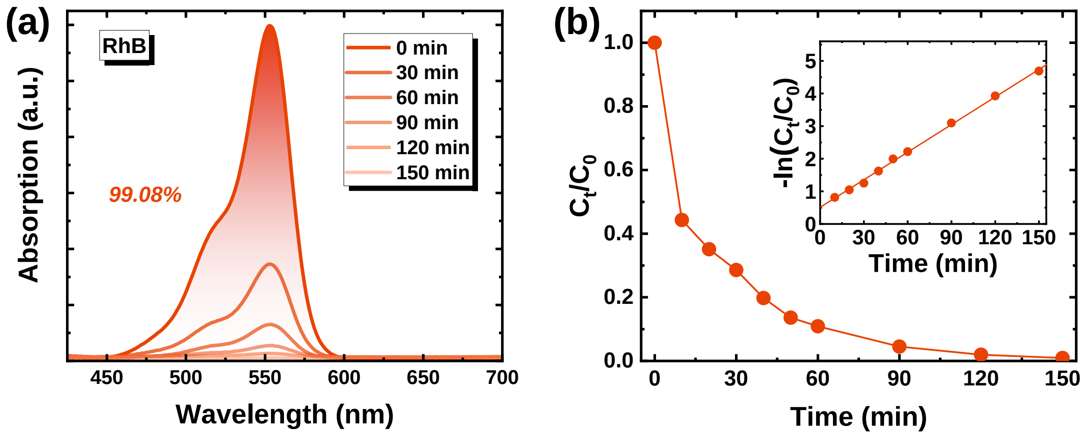

Figure S11. The absorption spectra of the photocatalytic degradation of (a) RhB (50 mg/L, 20 mL) solution in the presence of PI-Cu2-xS films (4×5 cm2) under vis–NIR light irradiation for 2.5 hours, and (b) the corresponding degradation rate and kinetic curves (inset) with Figure 5b, c.

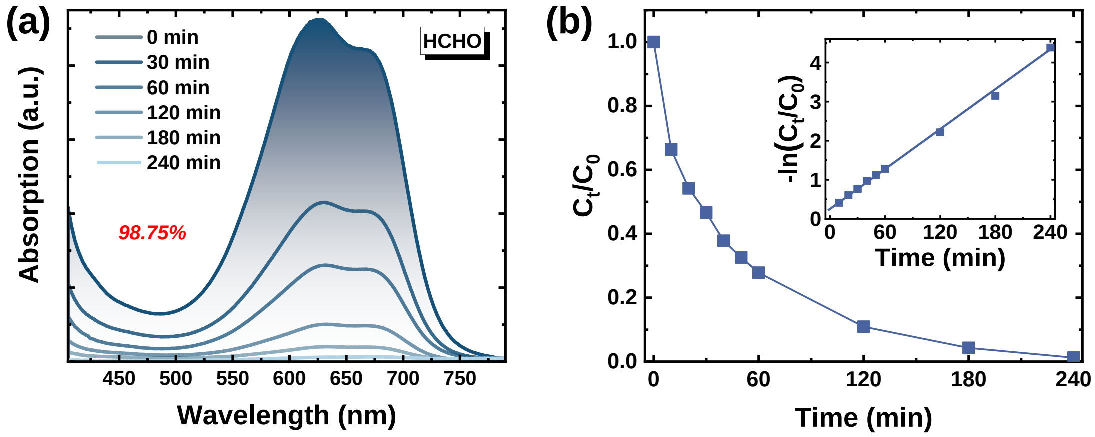

Figure S12. The absorption spectra of the photocatalytic degradation of (a) HCHO (50 mg/L, 20 mL) solution in the presence of PI-Cu2-xS films (2.5×4 cm2) under vis–NIR light irradiation for 4 hours, and (b) the corresponding degradation rate and kinetic curves (inset) with Figure 5b, c.

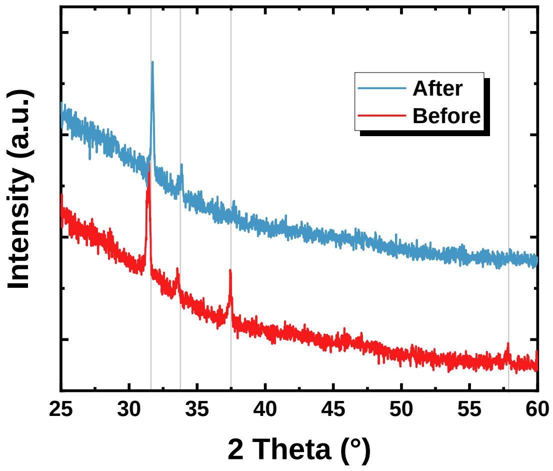

Figure S13. XRD comparison diagram between original and after 4 cycles of testing samples.

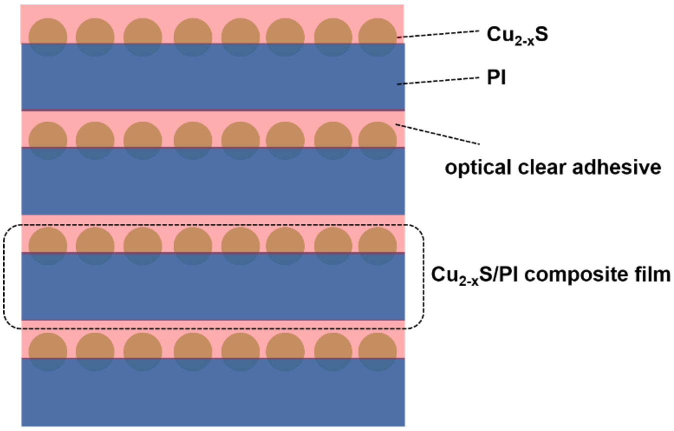

Figure S14. Schematic illustration of improved service life of composite films by the layer by layer method.
